# Public health workforce survey data (2016–2021) related to employee turnover: proposed methods for harmonization and triangulation

**DOI:** 10.3389/fpubh.2023.1306274

**Published:** 2024-01-05

**Authors:** Nicole M. Weiss, Skky Martin, Sezen O. Onal, Nicole McDaniel, Jonathon P. Leider

**Affiliations:** Center for Public Health Systems, Division of Health Policy and Management, University of Minnesota School of Public Health, Minneapolis, MN, United States

**Keywords:** public health systems, workforce, turnover, triangulation, harmonization, survey data

## Abstract

**Introduction:**

Public health workforce numbers are unsustainable at best and dire at worst: based on 2017 and 2019 data, 80,000 FTEs needed to be hired by health departments to provide basic public health foundational services *before* COVID-19 hit, suggesting that the situation is worse after the mass exodus of public health officials due to the pandemic. As such, a better understanding of public health workforce turnover is critical to improving recruitment and retention in the discipline.

**Methods:**

This methods report details how the authors harmonized four public health workforce surveys—the Public Health Workforce Interests and Needs Survey (PH WINS), the National Association of County and City Health Officials (NACCHO) Profile, the NACCHO Forces of Change survey, and the Association of State and Territorial Health Officials (ASTHO) Profile—in order to examine employee turnover.

**Results:**

We found that 31% of the public health workforce reported considering leaving their positions at some time in the future. Furthermore, the majority of agencies reported that zero vacancies had been filled in both 2018 and 2019.

**Discussion:**

These findings suggest that retention, recruitment, and onboarding may be areas upon which to focus evaluation and quality improvement endeavors, allowing public health organizations to better attract and retain the most qualified candidates.

## 1 Introduction

Despite its mission to enhance the health of the population nationwide, the American public health workforce has historically been understaffed (1–3). More current numbers prove no more optimistic. Estimates of the public health workforce numbered around 500,000 in 1980, dropping to 448,000 in 2000, and dropping again to 291,000 in 2014 (4). Leider et al. noted that ~15–20% of the public health workforce left the discipline during the Great Recession alone (5). More recent numbers are as of yet unavailable, though the Consortium for Workforce Research in Public Health—comprising scholars from the University of Minnesota, Columbia University, East Tennessee State University, Indiana University, Johns Hopkins University, and the University of Washington—are currently working on an enumeration of the public health workforce and will repeat the endeavor in 5 years' time to examine the effect of the COVID-19 pandemic (and pandemic funding) on workforce numbers.

Though we do not yet know precisely how COVID-19 has affected the public health workforce numbers, survey data from 2021 showed that a staggering 44% of governmental public health employees reported that they were considering leaving their jobs in the next 5 years (this includes those who were planning on retiring) (6). Furthermore, prior to COVID-19, 80,000 full-time equivalents (FTEs) needed to be hired by health departments to provide public health foundational services (that is, for public health to operate at the absolute minimum capacity to manage population health) (5). As it stands, public health workforce numbers are unsustainable at best and dire at worst.

As such, a better understanding of public health workforce turnover is critical to improving recruitment and retention in the discipline, and multiple surveys seek to provide these data for public health systems researchers (4, 7, 8). The Public Health Workforce Interests and Needs Survey (PH WINS) reports on an individual level and provides information on workforce demographics as well as intent to leave and other related variables (9, 10). Other surveys such as the National Association of County and City Health Officials (NACCHO) Profile, the NACCHO Forces of Change (FOC) survey, and the Association of State and Territorial Health Officials (ASTHO) Profile provide similar information but on an agency-level scale, meaning that one representative of an organization fills out the survey on behalf of the entire agency to provide that organization's data (11–13).

Comparing findings from these surveys is complicated by the surveys having been developed at different times, by different organizations, and for different aggregation levels. However, such a comparison is necessary to gain a more accurate understanding of public health employees' choices to remain in or leave their jobs. Before such a comparison can be attempted, dataset harmonization and triangulation must be completed. Harmonization is the process of adjusting different datasets to make them comparable, allowing researchers to aggregate or compare findings across these harmonized datasets (14). Triangulation, though related to harmonization, involves cross-verifying findings across different datasets, methodologies, or theoretical approaches (8).

It is essential to emphasize that workforce readiness is not predetermined solely by workforce numbers. Though we acknowledge that worker competencies also play a significant role in workforce readiness, a comprehensive examination of competencies is beyond the scope of this manuscript, as we only focus on workforce numbers. This methods report builds upon the foundation of prior harmonization work (14–17) and details how the authors harmonized and triangulated data from recent iterations of PH WINS (2021), the NACCHO Profile (2019), the NACCHO FOC survey (2020), and the ASTHO Profile (2019). We later describe the results of our harmonization and triangulation, focusing on variables related to turnover [including both voluntary and involuntary leave, which have different triggers (18)], and we also detail the limitations of our current work. Lastly, we discuss implications of these findings for public health systems research and practice, and we propose that further research be done to expand upon the methods presented here.

## 2 Materials and methods

### 2.1 Harmonization and triangulation

Data from PH WINS, the NACCHO Profile, the NACCHO FOC survey, and the ASTHO Profile were used to investigate public health workforce turnover. Each survey had different samples and numbers of responses. Around 30,000–40,000 responses was the average for the PH WINS items used in our analyses. Analogous numbers for the other surveys were about 1,500–5,000 responses for the NACCHO Profile, 500 for the NACCHO FOC survey, and 50 for the ASTHO Profile.

PH WINS data were collected in 2021 but describe the governmental (city/county and state/territorial) public health workforce over the years of 2018–2021. NACCHO Profile data were collected in 2019 and describe the city and county health department workforce for that same year (unless survey questions specifically asked about prior years); likewise, the NACCHO FOC survey was distributed in 2020, and its data reflect the city and county health department workforce for 2020 (unless survey questions specifically asked about prior years). The ASTHO Profile data were collected in 2019 but describe the state and territorial health agency workforce in the following fiscal years: 2016, 2017, and 2018. Because some data were collected before the COVID-19 pandemic and some data were collected at the beginning of the pandemic, results should be interpreted with caution. We expand upon this further in the limitations section below.

Though all surveys have been conducted over multiple iterations (e.g., PH WINS has been fielded in 2021, 2017, and 2014), we focus our analyses on the most recent iterations of these surveys (PH WINS 2021; NACCHO Profile 2019; NACCHO FOC 2020; ASTHO Profile 2019). We do bring in some statistics from prior survey iterations for context, but a comprehensive comparison of results over time is outside the scope of this manuscript.

The authors first examined the surveys' codebooks, identifying data themes during this period of “data discovery.” The data themes were: (1) demographics, (2) leave, (3) retire, (4) stay, and (5) hire. Survey questions that fit these themes were then identified and listed in a spreadsheet, which included information on the original survey question, the original code label, and the original code value. For example, any items in the four codebooks listed above that were related to the theme of “leaving”—such as “Are you considering leaving your job in the next 5 years?”—were included in the “leave” data theme. The exception to this was questions about leaving that were specifically about retiring; these were included in the “retire” data theme.

Harmonization and triangulation of survey questions regarding demographic characteristics was limited because the NACCHO Profile examined demographic information for top executives only, while PH WINS and the ASTHO Profile looked at demographic information for the workforce more broadly. Because these samples were not comparable, we focused our harmonization of demographic variables on PH WINS and ASTHO.

**Figure 1:**
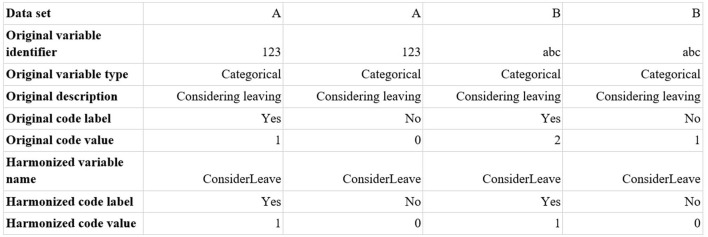
Generic example of harmonization protocol.

Questions within each of the data themes were then compared to find similar questions that had been asked in different surveys (Figure 1). A harmonized description of the survey question, as well as a harmonized code label and a harmonized code value, were added to the spreadsheet to indicate that items had been harmonized. Code value schemas were standardized (e.g., No = 0; Yes = 1). Data themes were originally assigned to one team member; they were then checked by another team member for quality control.

### 2.2 Descriptive and inferential statistics

Descriptive statistics were then computed for all harmonized variables to allow for comparison between the resulting values (14–17). More specifically, we created frequency/percentage tables tabulated at the agency level for the NACCHO Profile, NACCHO FOC, and the ASTHO Profile. We created frequency/percentage tables for PH WINS tabulated at the individual level because PH WINS responses were from individuals (not representative of an agency). We also calculated summary statistics, including the mean, standard deviation, minimum value, median, maximum value, and the 25th and 75th percentiles. All descriptive statistical calculations and linear probability regressions were performed in Stata 17 (19).

## 3 Results

The following describes results by data theme, including (1) demographics, (2) leave, (3) retire, (4) stay, and (5) hire.

### 3.1 Demographics

PH WINS demographic data are reported as proportions/percentages of the entire survey sample population because PH WINS data are collected on an individual level. Because ASTHO data are agency level, ASTHO demographic data are reported as averages of all the agencies' responses (i.e., an average of an average).

Of the race and ethnicity category options participants could choose from Table 1, those that are underrepresented in the public health workforce [compared to the general American population in 2021 (20)] are: Hispanic/Latino (PH WINS 17.96%; ASTHO mean 7.42; Census 18.86%) and white (PH WINS 53.72%; ASTHO mean 73.79; Census 75.73%) (the majority of participants in both surveys are white). Native Hawaiians and Pacific Islanders—the least represented overall in the public health workforce—are overrepresented in public health (PH WINS 0.38%; ASTHO mean 0.71; Census 0.26%). People who identify as Black or African American (PH WINS 15.33%; ASTHO mean 14.71; Census 13.58%), as well as those who are more than two races (PH WINS 4.30%; ASTHO mean 5.11; Census 2.96%), are also overrepresented in public health compared to the general population. Indigenous or Native (PH WINS 0.94%; ASTHO mean 1.64; Census 1.30%) as well as Asian (PH WINS 7.36%; ASTHO mean 5.46; Census 6.17%) folks are represented at roughly the same proportion in the public health workforce as in the general population.

**Table 1:** Demographics of survey participants (PH WINS and ASTHO Profile data).

	**PH WINS**	**ASTHO Profile**	
	**Percent**	**Mean**	**SD**
**Race/ethnicity**			
Asian	7.36	5.462	9.429998
Black or African American	15.33	14.708	16.6549
Native or Indigenous	0.94	1.636	1.83945
Native Hawaiian or Pacific Islander	0.38	0.71	1.525464
White	53.72	73.794	19.66546
Two or more races	4.3	5.114	4.767664
Hispanic or Latino	17.96	7.422449	9.669718
Not Hispanic or Latino	82.04	92.44286	9.619381
**Gender**			
Non-binary/other	1.77	0.386	0.4575935
Female	78.56	74.946	6.966163
Male	19.66	24.666	7.031753
**Age**			
Average age	45.73359	47.34898	1.810286
Median age	46	48.07083	3.266331
25th percentile	36	N/A	N/A
75th percentile	56	N/A	N/A
**Service**			
Years of service	11.95235	10.40204	1.780741

According to ASTHO respondents, women comprise on average three-quarters of each agency's workforce, men comprise about a quarter, and non-binary and other genders comprise < 1%. Women are also overrepresented in the workforce according to PH WINS respondents, numbering at about 79% (men are roughly 20%, and other genders are ~2%). Both surveys reported average ages of employees as around 45. In addition, participants of both surveys reported average years of service around 10–12 years.

### 3.2 Leave

#### 3.2.1 Voluntary leave

PH WINS data are reported at the individual level. According to PH WINS, in 2021 ~27% of public health employees were considering leaving their positions in the next year (see Section 3.4 for more information on how COVID-19 affected this number). Roughly 31% were considering leaving in general (no timeframe given), with about 5% planning to retire, 7% planning to take another job in government public health, 5% planning to take another government job *not* in public health, 4% planning to take another *non*-government public health job, 6% planning to take another *non*-government job *not* in public health, 2% planning to pursue further education, and 1% planning to leave the workforce. In sum, nearly a third of public health employees reported that they were considering leaving their jobs at some point in the future.

According to the ASTHO Profile survey that was conducted in 2019, which focused on state and territorial health agencies, the average number of public health employees by state health agency (SHA) who left their jobs slightly decreased from fiscal year (FY) 2016 (~318) to fiscal year 2018 (~296) (Table 2). This is also true for the median during this same time period (176 in FY 2016 compared to 147 in FY 2018). The maximum number of employees at one agency who left their jobs in FY 2016 was 3,729; in FY 2017, this number was 3,719. In FY 2018, this number jumped to 3,949.

**Table 2:** Summary statistics for separations at the agency level (ASTHO Profile data).

	**Separated FY 2016**	**Separated FY 2017**	**Separated FY 2018**
Mean	317.7111	291.5435	295.7447
SD	566.0043	554.7711	582.3767
Min	12	16	26
p25	61	63	55
p50	176	157.5	147
p75	369	288	307
Max	3,729	3,719	3,949

According to the NACCHO Profile (conducted in 2019) and the NACCHO FOC survey (conducted in 2020), which both focused on county and city health agencies, on average per agency 0.79 employees were lost through attrition in 2018 (number of responding agencies = 1,451), with 0.48 employees lost through attrition in 2019 (number of responding agencies = 549). On average, smaller agencies (>50,000) lost fewer employees (15%) compared to medium (50,000–499,999) and larger (500,000+) agencies, 44 and 45%, respectively.

#### 3.2.2 Involuntary leave

Also according to the NACCHO Profile and the NACCHO FOC survey, on average per agency 0.29 employees were laid off in 2018 and 0.14 employees were laid off in 2019. Put another way, only 107 agencies out of 1,450 total agencies laid off at least one employee in 2018. 1,450 agencies provided responses for data collected in 2019, while 545 agencies provided responses for data collected in 2020 (data collected in 2019 reflect 2018 numbers, and data collected in 2020 reflect 2019 numbers). On average, smaller agencies (>50,000) laid off fewer employees (6%) compared to medium (50,000–499,999) and larger (500,000+) agencies, 39 and 74%, respectively.

Fewer than 0.1 employees per agency on average saw their hours reduced or were furloughed in both 2019 and 2020 (number of responding agencies = 1,452 and 546, respectively). These data suggest that involuntary turnover at these agencies was low on average across all agencies regardless of the size of the population served. A slightly different story emerges when examining layoffs while taking into account the size of the constituent population, with mean layoffs per 100,000 population tending to be higher at agencies that serve smaller numbers of constituents (Table 3).

**Table 3:** Layoffs by 100 k population served (NACCHO Profile and NACCHO FOC data).

	**2018 Layoffs (NACCHO Profile)**			**2019 Layoffs (NACCHO FOC)**		
**Population count**	***N***	**Mean layoffs per 100 k pop**	**Mean layoffs**	***N***	**Mean layoffs per 100 k pop**	**Mean layoffs**
< 25 k	27	16.62952	1.592593	6	22.49274	3
25–49 k	20	5.618003	2.1	5	23.41459	8.6
50–99 k	8	3.764231	2.5	2	2.068731	1.5
100–249 k	19	3.820153	5.684211	2	1.941054	3
250–499 k	14	1.763976	6.714286	3	0.5972061	2
500–999 k	9	1.006315	8	0	N/A	N/A
1 million+	10	0.2222346	4.5	1	0.0674983	1

Also, it is important to note that NACCHO Profile and NACCHO FOC survey data do not cover layoffs for cause or other reasons for involuntary leave.

#### 3.2.3 Linear probability model

In our linear probability model (built using PH WINS data) (Table 4), we found several associations between race, gender, age, and the likelihood of individuals considering leaving. It's important to emphasize that the relationships identified are associative and do not imply causality, so results should be interpreted with caution.

**Table 4:** Associations between race, gender, age, and the likelihood of individuals considering leaving (PH WINS data).

	**Coefficient**	**Standard error**	***t***	***P* > *t***	**(95% conf. interval)**	
Native or Indigenous	0.0381992	0.0296245	1.29	0.197	−0.0198657	0.0962642
Asian	−0.0088544	0.0112429	−0.79	0.431	−0.030891	0.0131821
Black or African American	0.013953	0.0082739	1.69	0.092	−0.0022642	0.0301701
Hispanic or Latino	−0.0083577	0.0078571	−1.06	0.287	−0.0237578	0.0070424
Native Hawaiian or Pacific Islander	−0.0819618	0.0344828	−2.38	0.017	−0.1495492	−0.0143745
Two or more races	0.0921937	0.0155816	5.92	0	0.0616532	0.1227341
Man	0.0554625	0.0073416	7.55	0	0.0410727	0.0698523
Non-binary or other	0.1608011	0.0257287	6.25	0	0.110372	0.2112303
< 21 years	0.0904639	0.0582247	1.55	0.12	−0.0236582	0.2045861
21–30 years	0.1263144	0.010289	12.28	0	0.1061476	0.1464812
31–40 years	0.0642224	0.008108	7.92	0	0.0483305	0.0801142
51–60 years	−0.0576598	0.0074971	−7.69	0	−0.0723542	−0.0429653
>61 years	−0.1304018	0.0089408	−14.59	0	−0.147926	−0.1128776
Coefficient of constant variable	0.2431428	0.0061766	39.37	0	0.2310365	0.2552492

References: (1) “white” for race, (2) “41–50” for age, and (3) “women” for gender.

Compared to those identifying as white, there are differences among ethnic groups in the likelihood of considering leaving. Native Hawaiian or Pacific Islanders have a notable reduction in this likelihood by 8.2 percentage points (*p* < 0.05). In contrast, individuals of two or more races show an increased probability by 9.2 percentage points (*p* < 0.01). Black or African Americans also show an increase by 1.3 percentage points, though this is only marginally significant (*p* < 0.1). The variations seen in those who identify as Asian, Hispanic or Latino, and Native or Indigenous—reductions by 0.09 percentage points (*p* = 0.43), 0.09 percentage points *(p* = 0.28), and an increase of 3.8 percentage points (*p* = 0.19), respectively—are not statistically significant.

Regarding gender, compared to those categorized as women, men were associated with a 5.5 percentage point increase (*p* < 0.01) in the likelihood of considering leaving, while non-binary or other had a 16.1 percentage point increase (*p* < 0.01).

When considering age as a factor, individuals in the 21–30 and 31–40 years of age categories show increased probabilities of considering leaving compared to those aged 41–50 years. Specifically, the likelihood increases by 12.6 and 6.4 percentage points for the respective age groups (all *p* < 0.001). In contrast, individuals aged above 61, along with the 51–60 age group, exhibit a significant decrease in this likelihood, with a reduction of 13 (*p* < 0.001) and 5.76 (*p* < 0.001) percentage points, respectively. While individuals under 21 exhibit a 9.0 percentage point increase in the likelihood of considering leaving when compared to the 41–50 age group, this difference is not statistically significant (*p* = 0.12).

In sum, no clear trends emerge from the harmonization of leave data, though the average and median numbers of employees who left their jobs at state or territorial agencies decreased over time, as did the average numbers of those at city and county agencies who were laid off or saw positions lost through attrition.

### 3.3 Retire

ASTHO Profile responses from 2019 indicate that on average at the state and territorial level, ~15 employees per agency would be eligible for retirement in FY 2019 and FY 2020, roughly 17 would be eligible for retirement in FY 2021, around 18 would be eligible in FY 2022, and ~19 would be eligible in FY 2023 (Table 5). The median number eligible for retirement also increased during this time period, from 14 in FY 2019 to 19 in FY 2023. This is also true for the maximum number of people reported by one agency who would be eligible for retirement, which was 51 in FY 2019 and 61 in FY 2023. The standard deviation also increased from 11.50 in FY 2019 to 14.71 in FY 2023.

**Table 5:** Summary statistics for retirement eligibility (ASTHO Profile data).

	**Retire eligible FY 2019**	**Retire eligible FY 2020**	**Retire eligible FY 2021**	**Retire eligible FY 2022**	**Retire eligible FY 2023**
Mean	15.46267	15.07891	16.55533	17.965	19.30978
SD	11.49954	12.20849	13.18258	14.00826	14.70828
Min	0	0	0	0	0
p25	8	5.5	6.9	5	8
p50	14	13.33	15	16.5	19
p75	20	18.5	20.6	23.7	26.7
Max	51	53	55	58	61

At the more local level, NACCHO Profile data reveal that, on average, about two employees retired in 2019 per agency while roughly the same number planned to retire in the following year. However, the standard deviation more than doubled from 3.27 in 2019 to 6.95 in 2020. And the maximum number of employees who retired in 2019 that was reported by one agency (20) was just under one-sixth of the maximum number of employees who planned to retire in 2020 reported by one agency (115).

According to the PH WINS individualized data, roughly 36% of the workforce has taken steps to retire (mean age of those who have taken steps was roughly 63). Furthermore, ~20% of respondents were considering retiring in the next 5 years (mean age of those who were was ~60), with about 5% of total respondents indicating they planned to retire within 2 years. Unfortunately, we do not have specific data on how COVID-19 has affected these numbers.

In sum, one-fifth of public health workers surveyed planned to retire within 5 years, and the number of state and territorial agency employees planning to retire increased over time. This suggests that public health will be considerably impacted by the mass retirements of the baby boomer generation.

### 3.4 Stay

The only dataset containing questions about remaining at an organization or agency was PH WINS; though triangulation and harmonization are not possible with only one dataset, we have included results from this data theme to more fully understand the environment around employee turnover.

PH WINS individualized data from 2021 indicate that ~55% of respondents listed “job stability” as a primary reason for remaining at their jobs (Table 6). Nearly as popular was “flexibility (e.g., flex hours or telework),” with roughly 48% reporting this as a reason for staying. About 43% listed “job satisfaction,” and ~33% listed “exciting and challenging work” as primary reasons for remaining. Factors such as “unsatisfactory opportunities outside of the agency,” “lack of stress,” “satisfaction with your agency's leadership (e.g., Health Commissioner, Senior Deputy, etc.),” “acknowledgment/recognition for your work,” and “organizational climate/culture” did not appear to matter as much to PH WINS respondents, with only 8, 11, 18, 19, and 20%, respectively, saying that these reasons were primary motivators for remaining at their jobs.

**Table 6:** Reasons for remaining at job (PH WINS data).

	**No**		**Yes**	
	**Freq**.	**Percent**	**Freq**.	**Percent**
Acknowledgment/recognition for your work	25,314	81.23	5,850	18.77
Job satisfaction	17,794	57.1	13,370	42.9
Opportunities for advancement	26,028	83.52	5,136	16.48
Training opportunities	26,707	85.7	4,457	14.3
Satisfaction with your agency's leadership	25,688	82.43	5,476	17.57
Unsatisfactory opportunities outside of the agency	28,647	91.92	2,517	8.08
Pay	23,786	76.33	7,378	23.67
Satisfaction with your supervisor	17,112	54.91	14,052	45.09
Lack of stress	27,650	88.72	3,514	11.28
Flexibility (e.g., flex hours/telework)	16,175	51.9	14.989	48.1
Benefits (e.g., retirement contributions/pensions, health insurance)	10,535	33.81	20,629	66.19
Pride in the organization and its mission	18,453	59.21	12.711	40.79
Exciting and challenging work	20,744	66.56	10,420	33.44
Organizational climate/culture	25,077	80.47	6,087	19.53
Mentorship opportunities	29,171	93.6	1,993	6.4
Support	24,365	78.18	6,799	21.82
Job stability	14,092	45.22	17,072	54.78
Other (please specify)	24,848	92.57	2,316	7.43

Regarding COVID-19 specifically, about three-quarters of participants (76.4%) said that the pandemic did not impact their decision to leave or stay. About 15.5% said that COVID-19 made them want to leave, including (1) those who initially wanted to leave but wanted to leave *more* after COVID-19 (6.7%) and (2) those who initially wanted to stay but wanted to leave after COVID-19 (8.8%). Approximately 8.2% said COVID-19 made them want to stay, including (1) those who initially wanted to stay but wanted to stay *more* after COVID-19 (4.2%) and (2) those who initially wanted to leave but wanted to stay after COVID-19 (4.0%). Put simply, COVID-19 did influence the decisions of some workers to leave or stay, with the number who decided to leave almost double that of those who decided to stay. However, it should be noted that PH WINS 2021 data were collected between September 2021 and January 2022 and therefore reflect workers' feelings about the pandemic at that time. Their feelings may have changed as the pandemic continued and as reactions to public health became more polarized.

In sum, most survey respondents did not cite COVID-19 as the initial factor in making them want to stay or leave. Many workers emphasized the importance of job stability (i.e., knowing one will be employed for a long period of time) and flexibility (e.g., telework, flexible work hours) as reasons for staying.

### 3.5 Hire

Two surveys, the NACCHO Profile (conducted in 2019) and NACCHO FOC (conducted in 2020), examined hiring at an agency level (Table 7). Many local health departments (LHDs) reported in both these surveys that zero positions or vacancies were filled during 2018 and 2019. In 2018, for example, roughly 64% of agencies filled no positions (mean positions filled = 1.87), and ~95% of agencies said that the number of vacancies filled that year due to the lifting of previous hiring freezes was zero (mean positions filled by lifting hiring freeze = 0.15). These same figures for the following year were also roughly 64 and 95%, respectively (means = 2.12 and 0.19, respectively). Reported numbers for vacancies filled due to employee turnover were not as low. Roughly 35% of agencies filled zero positions in 2018 while about 41% filled one to four of these positions that same year (mean positions filled due to turnover = 7.27). In 2019, ~55% of agencies reported zero positions filled due to employee turnover; another 33% filled one to four of these positions (mean = 2.87).

**Table 7:** Summary statistics for hires (NACCHO FOC and NACCHO Profile data).

	**2018**			**2019**		
	**New positions filled**	**Filled due to lifting of hiring freeze**	**Filled due to turnover**	**New positions filled**	**Filled due to lifting of hiring freeze**	**Filled due to turnover**
Mean	1.869958	0.1468144	7.271399	2.119263	0.1863118	2.869646
SD	10.96945	1.60838	34.22918	8.273626	1.330765	8.38474

In sum, both the NACCHO Profile and the NACCHO FOC survey show that relatively few vacancies were filled in 2018 and 2019.

## 4 Discussion

Data triangulation and harmonization projects hold the potential to illuminate concordance and discordance within a field, especially around hard-to-measure constructs. In theory, quits, retirements, and separations generally should be among the more straightforward measures. Workforce data quality is a foundational issue in public health and the public sector more generally (14–17). However, researchers have shown that harmonization of disparate data sets and triangulation of these data sources is needed to answer the most fundamental questions (14–17), like how many people retire each year from public health. This is because surveys and administrative data sources and other means of collection go into the field for different reasons, using different instrumentation, at different points in time. So, naturally they yield different results. Ours is not the first project to find that harmonization is necessary to acquire usable field-wide estimates (14–17).

Approximately 27% of the state and local public health workforce reported considering leaving their positions in 2021, according to PH WINS data. Though not a completely analogous comparison, this number is slightly higher than the 20% of state health agency staff who reported that they were considering leaving in FY 2015 (when the 2017 PH WINS data were collected). A plausible factor contributing to this increase could be the COVID-19 pandemic. Of note, ~15% of the respondents to the 2021 PH WINS indicated that the pandemic incited thoughts of job resignation. Furthermore, PH WINS data from FY 2015 indicate that ~6% of state health agency workers planned to retire that year. This is similar to data from 2021 indicating that about 5% of total respondents planned to retire within 2 years. It should be noted that the survey items asked about 1- and 2-year timeframes, respectively, and results should be interpreted with caution. PH WINS respondents also indicated that the most important reasons for remaining at their jobs included job stability, flexibility (e.g., flex hours or telework), job satisfaction, and exciting and challenging work.

Much remains true about the workforce as before COVID entered the fray. Stress and burnout are important reasons for leaving. However, staff fundamentally considered leaving because of a lack of engagement, a lack of a path forward at their agency, and a lack of supervisory satisfaction. These factors were considered as much or more than pay dissatisfaction as reasons for leaving, meaning they should be prioritized by managers and supervisors who seek to build a strong work environment. Human resources professionals should seek to address these factors during recruitment efforts to encourage the best candidates to apply for open positions. However, it is important to note that there is no one-size-fits-all solution to recruitment; emphasizing one contextual factor may work for one agency's recruitment needs but not for another (21, 22).

One of the main takeaways from the NACCHO Profile and NACCHO FOC data from 2018 and 2019 is that many agencies filled zero positions or vacancies during these years immediately prior to COVID-19. This suggests ongoing difficulty in recruiting qualified candidates, something that may be addressed by highlighting in job postings the most important reasons for remaining at jobs as described above—and in particular, how that job epitomizes those factors. Despite high levels of job vacancies, retirement eligibility, and a need to grow the workforce, the field was unable to fully staff up, suggesting that staffing strategies were unsustainable (21). Now that we know all too well what an underbuilt workforce means for pandemic readiness and response, more efforts can and should be considered around recruitment competitiveness for local health departments. These efforts should be tailored separately for rural jurisdictions and big cities, which face very different labor market pressures (23).

Of note is recent growing interest in public health as an undergraduate major. The number of undergraduate public health programs has grown substantially over the past 20 years, and the number of undergraduate degrees awarded has outpaced the number of master's degrees awarded in public health (24). Despite increasing numbers of graduates with training in public health, agencies still struggle to fill positions. Possibly contributing to this are long, arduous interviewing and onboarding processes for public service jobs (25, 26) and salaries and benefits that are unmatched by those offered by the private sector (27). Future research should examine this trend to determine why public health graduates are seeking employment elsewhere than in governmental public health.

### 4.1 Limitations

This work harmonizes data from PH WINS, the NACCHO Profile, the NACCHO FOC survey, and the ASTHO Profile. However, this is not an all-inclusive list of surveys that provide information on the public health workforce. Examples of such surveys include but are not limited to the Association of Public Health Laboratories' Laboratorian Workforce Survey, the Council of State and Territorial Epidemiologists: Epidemiology Capacity Assessment, the Association of Schools and Programs of Public Health Annual Reporting, and the Centers for Disease Control and Prevention (CDC) and ASTHO Workforce Gaps Survey. Time constraints prevented the author team from being able to incorporate data from these surveys for this work, but the authorship group hopes to study these aforementioned surveys, along with multiple others, on a larger-scale harmonization and triangulation project in the future.

Furthermore, the different time frames during which the surveys were conducted will influence our results' generalizability. For example, the most recent PH WINS data were collected in 2021; in contrast, the most recent NACCHO Profile and ASTHO Profile data that were used in these analyses were collected in 2019. More specifically, some of the data were collected before the COVID-19 pandemic, and some of the data were collected early during the pandemic. This may have influenced both the responses given specifically and the state of the workforce more broadly. In a similar vein, questions about COVID-19 were added to the 2021 PH WINS survey as well as the 2020 NACCHO FOC survey, but COVID-19-related questions did not appear in the 2019 ASTHO Profile nor in the 2019 NACCHO Profile.

In addition, we only examine one component of workforce readiness (workforce numbers) in this article. Our results do not speak to workforce competencies, which also is a critical component of workforce readiness.

## 5 Conclusions

Given that nearly one third of the current public health workforce is considering leaving their jobs at some time in the future (leaving timeline was not specified in this particular PH WINS 2021 survey item), historic trends of understaffing in public health do not appear to be changing imminently. It is yet unknown whether increased interest in public health in light of the pandemic will be sustained, and the imminent retirement of many in the baby boomer generation also poses challenges for the public health workforce.

To best prepare to meet these challenges, further research on turnover and retention is needed. This work should build upon the harmonization and triangulation methods that have been presented here, expanding to other data themes that affect workforce readiness. This future work should also translate these findings into policy to streamline the governmental public health hiring process. Only by properly staffing our public health workforce can we transform public health from a field that reacts to threats into a field that prevents them.

## Data availability statement

Publicly available datasets were analyzed in this study. This data can be found here: https://debeaumont.org/phwins/2021-findings/; https://www.naccho.org/resources/lhd-research/national-profile-of-local-health-departments; https://www.naccho.org/resources/lhd-research/forces-of-change; https://astho.org/profile/.

## Ethics statement

The studies involving humans were approved by University of Minnesota Institutional Review Board. The studies were conducted in accordance with the local legislation and institutional requirements. Written informed consent for participation was not required from the participants or the participants' legal guardians/next of kin in accordance with the national legislation and institutional requirements.

## Author contributions

NW: Conceptualization, Data curation, Formal analysis, Investigation, Methodology, Project administration, Resources, Supervision, Validation, Visualization, Writing – original draft, Writing – review & editing. SM: Conceptualization, Data curation, Formal analysis, Investigation, Methodology, Project administration, Resources, Supervision, Validation, Visualization, Writing – review & editing. SO: Conceptualization, Data curation, Formal analysis, Investigation, Methodology, Validation, Visualization, Writing – review & editing. NM: Data curation, Formal analysis, Investigation, Methodology, Writing – review & editing. JL: Conceptualization, Data curation, Funding acquisition, Investigation, Methodology, Project administration, Resources, Supervision, Validation, Visualization, Writing – original draft, Writing – review & editing.
